# Novel Electrochemical Sensing Strategy for Organophosphorus Pesticide Residues

**DOI:** 10.3390/bios16040208

**Published:** 2026-04-07

**Authors:** Mingzhuo Xu, Chen He, Jiajing Zhang, Hao Yang, Xiuzhong Wang

**Affiliations:** 1 College of Chemistry and Pharmaceutical Sciences, Qingdao Agricultural University, Qingdao 266109, China; 20230201146@stu.qau.edu.cn (M.X.); 20230201092@stu.qau.edu.cn (J.Z.); 20230201103@stu.qau.edu.cn (H.Y.); 2 Shandong Provincial Metrology and Testing Center, Jinan 250102, China; hechenmacao@126.com

**Keywords:** electrochemical biosensor, pesticide pesticides, isazofos, food safety

## Abstract

Organophosphorus pesticide residues (OPPs) pose significant threats to ecological systems and human health, and conventional detection techniques are cumbersome, time-consuming, and costly. Herein, a facile electrochemical biosensor has been constructed based on a methyl green/chitosan (MG/Chi) composite membrane-modified electrode for the selective detection of OPPs, using isazofos (Isa) as the model analyte. Experimental results demonstrated that Isa significantly decreases the redox peak current of the modified electrode in buffer solution, and a good linear relationship was observed between the change in peak current and Isa concentration within a specific range. This biosensor exhibits excellent anti-interference capability and high sensitivity, with a limit of detection (LOD) as low as 0.60 μM. Furthermore, it was successfully applied for the quantitative determination of OPPs in real food and environmental samples, which confirms its reliable practical applicability and potential for on-site monitoring.

## 1. Introduction

Modern agriculture is highly dependent on pesticide application to secure crop yields and mitigate biotic stresses. Owing to their remarkable insecticidal efficacy, organophosphorus pesticides (OPPs) account for approximately 30% of the global pesticide market [1,2]. However, the relative persistence of OPPs leads to their accumulation in soil, water, and food matrices, posing severe threats to ecological balance and human health via the food chain [3,4,5]. OPPs exert neurotoxic effects by irreversibly inhibiting acetylcholinesterase (AChE), disrupting cholinergic signaling and inducing acute or chronic health disorders [6,7,8,9]. This widespread risk underscores the urgent need for accurate, rapid, and on-site detection methods for OPP residues, which is critical for environmental monitoring and public health protection [10,11,12].

Numerous analytical techniques have been developed for OPP detection, including gas chromatography (GC), high-performance liquid chromatography (HPLC), and gas chromatography–mass spectrometry (GC-MS) [13,14,15]. These chromatographic methods offer high separation efficiency and detection accuracy but are limited by cumbersome sample pretreatment, expensive instrumentation, long analysis cycles, and reliance on professional operators [16,17]. Immunoassays and enzyme inhibition assays provide simpler operation but suffer from drawbacks such as labor-intensive antibody preparation, poor enzyme stability, stringent reaction conditions, and high costs, restricting their on-site application [18,19,20,21,22,23]. Accordingly, the development of low-cost, label-free, and field-deployable detection strategies still constitutes a critical challenge in this field.

Biosensors have emerged as a promising alternative due to their simplified configuration, rapid response, and high sensitivity [24,25]. Optical biosensors (e.g., colorimetric, fluorescent, surface-enhanced Raman spectroscopy-based) have demonstrated excellent detection performance, but most require complex probe labeling, increasing operational complexity and cost [26,27,28]. For instance, Hu et al. [29] developed an aptamer-based fluorescence resonance energy transfer sensor with gold nanoparticle sensor for OPPs in edible mushrooms, while Cai et al. [30] reported an aggregation-induced emission fluorescent and colorimetric biosensor to detect OPPs with a detection limit of 0.3 μg/L. Despite substantial progress in this field, the scarcity of simple, label-free, and cost-effective sensing platforms severely impedes their practical translation and large-scale application.

Electrochemical sensors have garnered increasing attention for the detection of OPPs, owing to their inherent advantages, including outstanding miniaturization potential, low cost, and capability for real-time in-field analysis [31]. Electrochemical sensors for OPPs are generally classified into direct and indirect methods. Compared with direct electrochemical detection, indirect electrochemical sensors offer several distinct advantages [32]. Specifically, they circumvent the direct redox reaction of target pesticides on the electrode surface, which effectively mitigates electrode fouling and thereby improves the long-term stability of detection [33]. More importantly, this strategy enables highly selective sensing of non-electroactive or poorly electroactive pesticides, which are challenging to quantify via direct electrochemical approaches. However, indirect electrochemical sensors also present inherent limitations. Their sensing signals are highly dependent on intermediate reactions, which necessitates rigorous control over experimental conditions including pH, temperature, and incubation time. Meanwhile, coexisting components in real sample matrices may introduce non-negligible interference, and the fabrication of the required sensing interfaces is generally more complex [34]. Accordingly, direct electrochemical detection has been more widely adopted for OPP analysis. For instance, Qin et al. [35] achieved ultrasensitive detection of paraoxon (limit of detection, LOD: 17 pg/mL) using a dissolvable photoelectrode, while Shalini et al. [36] developed a glassy carbon electrode (GCE) modified with carbon black for triazophos detection, achieving an LOD of 119 pg/mL. Despite considerable progress in direct voltammetric sensing of organophosphorus pesticides [37], the reported sensing platforms still face bottlenecks for isazofos detection, including complicated fabrication processes, narrow linear detection ranges, and insufficient field applicability. For example, the Zirconia nanocomposite sensor developed by Liu et al. [38] achieves an ultra-low LOD but requires tedious material synthesis and multi-step electrode modification. The AI-powered multimodal robotic sensing system (M-Bot) proposed by Yu et al. [39] enables efficient traceability of trace toxicants, yet it depends on sophisticated fabrication infrastructure and high costs. In general, most existing modified electrodes suffer from complex preparation procedures or insufficient specificity, which severely hinders their practical promotion and application [40].

Methyl green (MG), a water-soluble cationic dye with excellent chemical stability that is widely used as a DNA stain in chromogenic assays [41,42], has rarely been explored for its electrochemical properties and application potential in pesticide detection to date. Cyclic voltammetry (CV) characterizations have confirmed that MG exhibits a well-defined redox peak at ~0.42 V, which originates from its quinoid moiety [43]. This redox-active structure can specifically interact with certain OPPs, resulting in a measurable change in peak current, which lays the foundation for quantitative pesticide detection.

The main aim of this work is to construct a simple, label-free electrochemical biosensor based on a GCE modified with methyl green/chitosan (MG/Chi) composite film for sensitive and rapid detection of isazofos, a widely used OPP. The proposed MG/Chi composite-modified electrochemical sensor for direct isazofos detection addresses the aforementioned limitations, with three core advantages: (1) The sensor is fabricated via a one-step electrodeposition protocol, which avoids complex nanomaterial synthesis and tedious multi-step electrode functionalization. (2) The enzyme-free detection strategy provides a wide linear range of 0.002–2.5 mM with an LOD of 0.60 μM, which fully covers the practical residual limit requirements of isazofos. (3) The platform exhibits excellent anti-interference performance in complex real sample matrices, and it can be used as a low-cost, disposable platform for on-site rapid screening of isazofos.

## 2. Materials and Methods

### 2.1. Materials and Instruments

Methyl green and chitosan were purchased from Shanghai Lanji Technology Development Co., Ltd. (Shanghai, China). Glacial acetic acid, phosphoric acid, anhydrous ethanol, and methanol were purchased from Laiyang Kangde Chemical Co., Ltd. (Laiyang, China). Sodium hydroxide, disodium hydrogen phosphate, potassium chloride, potassium ferricyanide, potassium ferrocyanide, and calcium chloride were all purchased from Sinopharm Chemical Reagent Co., Ltd. (Shanghai, China). Isazofos, profenofos, diazinon, chlordane, dimehypo chlorpyrifos, parathion, paraoxon and glucose were purchased from Aladdin (Shanghai, China). Ultra-pure water was purified by the Millipore-Q system (18.2 MΩ cm^−1^).

We also used an ELMA E60H ultrasonic cleaner (Elma Schmidbauer GmbH, Singen, Germany), AR124CN electronic analytical balance (Ohaus Instruments (Shanghai) Co., Ltd., Shanghai, China), PB-10 pH meter (Sartorius Scientific Instruments Co., Ltd., Göttingen, Germany), 3 mm glassy carbon electrode, CHI 660E electrochemical workstation (Shanghai Chenhua Instrument Co., Ltd., Shanghai, China), FTIR-850 Fourier Transform Infrared (FTIR) spectroscope (Tianjin Gangdong SCI. & Tech. Co., Ltd., Tianjin, China), Dimension Icon Atomic Force Microscope (AFM) (Bruker, Karlsruhe, Germany), and Regulus8100 Scanning Electron Microscope (SEM) (Hitachi, Tokyo, Japan).

### 2.2. Experimental Methods

#### 2.2.1. Preparation of Chitosan Solution

Weigh 0.0500 g of chitosan powder and dissolve it in 1% (*v*/*v*) acetic acid solution to a final volume of 10.00 mL. Sonicate the mixture at 40 °C for 1 h to obtain a homogeneous chitosan solution.

#### 2.2.2. Preparation of Methyl Green/Chitosan (MG/Chi) Composite Solution

Dilute the stock methyl green solution (1 mg/mL) using the pre-prepared chitosan solution to obtain a series of MG/Chi composite solutions with different methyl green concentrations.

#### 2.2.3. Preparation of Isazofos Pesticide Solutions

To prepare the stock solution, accurately weigh 0.1000 g of isazofos into a 10.00 mL centrifuge tube, add 6.40 mL of methanol, and vortex vigorously until fully dissolved to yield a 50.00 mM isazofos stock solution. Serial dilutions of the stock solution were subsequently prepared using pH 7.5 phosphate-buffered saline (PBS) to achieve series of concentrations. Note: All isazofos working solutions should be prepared immediately prior to use to ensure stability.

#### 2.2.4. Preparation of Modified Electrode

The working electrode was sequentially polished with alumina (Al_2_O_3_) slurries of 0.1 μm and 0.03 μm particle sizes, then thoroughly rinsed with ultrapure water and anhydrous ethanol. After rinsing, the electrode was washed again with ultrapure water and air-dried at room temperature. Subsequently, a 5 μL aliquot of methyl green/chitosan (MG/Chi) composite solution was carefully drop-cast onto the electrode surface. The electrode was incubated undisturbed at room temperature for 3 h to enable complete solvent evaporation and uniform film formation, affording the as-prepared modified electrode for subsequent experiments.

#### 2.2.5. Electrochemical Measurements

All electrochemical measurements were performed using a conventional three-electrode configuration in a standard electrolytic cell. The working electrode was a glassy carbon electrode modified with the MG/Chi composite film, while a platinum wire served as the auxiliary electrode and an Ag/AgCl electrode (saturated KCl electrolyte) was employed as the reference electrode. The electrochemical behavior of the modified electrode was characterized using the following techniques:

(1) Cyclic Voltammetry (CV) Measurements

Cyclic voltammetry measurements were performed using glassy carbon electrodes modified with MG/Chi composite films of varying concentrations as working electrodes. All CV scans were conducted in a 100 μM isazofos solution under the following experimental conditions: potential range from 0 to 0.6 V (vs. Ag/AgCl), scan rate of 0.1 V/s, and a 2 s rest period between scans.

(2) Electrochemical Impedance Spectroscopy (EIS) Measurements

Electrochemical impedance spectroscopy (EIS) measurements were performed to characterize the interfacial properties of the modified electrode. Both the MG/Chi composite film-modified glassy carbon electrode and the bare glassy carbon electrode were used as working electrodes for comparison. The measurements were carried out in 1.0 mM Fe(CN)_6_^3−/4−^, with a biasing potential of 0.220 V, an amplitude of 5 mV and a frequency range of 1 Hz to 10 kHz.

(3) Differential Pulse Voltammetry (DPV) Measurements

Differential pulse voltammetry measurements were performed using a glassy carbon electrode modified with a 0.08 mg/mL MG/Chi composite film as the working electrode. DPV measurements were carried out in a series of isazofos solutions with gradient concentrations under the optimized experimental parameters: a potential window of 0.25–0.50 V (vs. Ag/AgCl), an enrichment time of 6 min, a pulse width of 0.05 s, a pulse amplitude of 50 mV, a step potential of 4 mV, and a scan rate of 10 mV/s.

#### 2.2.6. Assay of OPPs in Real Samples

To verify the practical applicability of the proposed sensing strategy, isazofos detection was performed in agricultural products (ginger and carrots) purchased from a local supermarket and natural water samples collected from the campus of Qingdao Agricultural University. For sample pretreatment, ginger and carrots were first chopped into small pieces, and their edible parts were homogenized using a juicer [44]. Twenty grams of the resulting homogenate was mixed with 20 mL of acetone, and the mixture was filtered through a 0.45 μm microfiltration membrane to eliminate insoluble impurities. Different volumes of an isazofos standard solution were individually spiked into the aforementioned extraction solution and natural water samples. The resulting solutions were then diluted to 10 mL prior to analysis. The spiked samples with isazofos concentrations of 20.00 and 100.00 μM were then analyzed, with six replicate measurements performed for each sample. DPV analysis was performed in a static solution without stirring.

## 3. Results and Discussion

### 3.1. Physicochemical Characterization of the MG/Chi Composite

**Figure 1:**
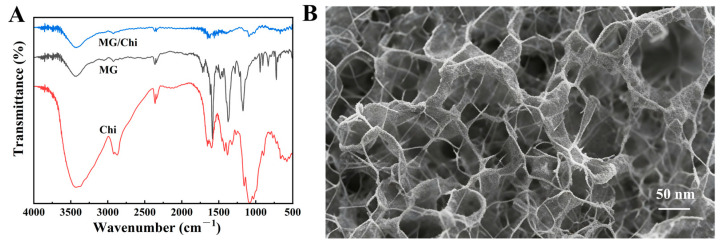
(**A**) Fourier transform infrared (FTIR) spectra of pristine Chi, pristine MG, and the MG/Chi composite film; (**B**) scanning electron microscopy (SEM) image of the MG/Chi composite film.

Fourier transform infrared (FTIR) spectroscopy was first performed to characterize pristine chitosan (Chi), pristine methyl green (MG), and the as-fabricated MG/Chi composite, with the corresponding spectra presented in Figure 1A. For pristine Chi, two characteristic absorption peaks were observed, at 3420 cm^−1^ (assigned to the stretching vibrations of -OH and -NH_2_ groups) and 1650 cm^−1^ (corresponding to the amide I band). For pristine MG, distinct absorption peaks appeared at 1580 cm^−1^ (aromatic ring skeletal stretching) and 1120 cm^−1^ (-SO_3_^−^ stretching vibrations). In the MG/Chi composite film, the -OH/-NH_2_ stretching peak of Chi shifted to 3435 cm^−1^, while the -SO_3_^−^ stretching peak of MG red-shifted to 1110 cm^−1^. These characteristic peak shifts serve as direct evidence for the formation of hydrogen bonding and electrostatic interactions between MG and Chi moieties, which endow the composite film with robust structural stability—an essential prerequisite for its application as an electrode modification material in sensing systems.

Subsequently, the surface morphological features of the as-prepared MG/Chi composite film on the GCE surface were investigated via scanning electron microscopy (SEM). As depicted in Figure 1B, the MG/Chi composite film forms a uniform, continuous, and interconnected porous structure on the GCE substrate, with a pore size distribution ranging from 50 to 100 nm. This well-defined porous architecture affords a large specific surface area for the efficient immobilization of MG (the electroactive core component of the sensing interface) and simultaneously accelerates interfacial electron transfer between the GCE substrate and the composite film—a critical process for enhancing the electrochemical signal response of the fabricated sensor. No clear agglomeration of MG nanoparticles or structural defects of the composite film were observed in the SEM micrograph, which verifies the excellent dispersibility of MG within the Chi matrix and the good film-forming property of the MG/Chi composite.

### 3.2. EIS Characterization of MG/Chi Composite Film-Modified GCE

Electrochemical impedance spectroscopy (EIS) was first employed to characterize the interfacial properties of the glassy carbon electrode (GCE) before and after modification. For the Nyquist plots derived from EIS measurements, the radius of the semicircular region exhibits a direct positive correlation with the charge transfer resistance (Rct) at the electrode/electrolyte interface.

**Figure 2:**
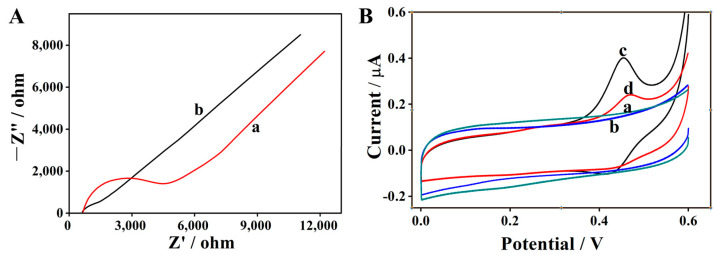
(**A**) EIS Nyquist plots of glassy carbon electrodes: (a) bare GCE; (b) MG/Chi composite film-modified GCE. (**B**) CV curves of the GCE in PBS (pH 7.50). (a) Bare GCE; (b) GCE + 100 μM isazofos; (c) MG/Chi composite film-modified GCE; (d) MG/Chi composite film-modified GCE + 100 μM isazofos.

As shown in Figure 2A, the bare GCE (curve a) exhibits a relatively large semicircle, which is indicative of a high Rct at its interface. In contrast, modification of the GCE with the methyl green/chitosan (MG/Chi) composite film (curve b) results in a dramatic reduction in the semicircle radius. This observation verifies that the MG/Chi composite film features excellent electrical conductivity, which expedites the electron transfer kinetics at the electrode/electrolyte interface. These results confirm successful modification of the MG/Chi composite film onto the GCE surface and highlight its superior electrochemical performance, which renders it highly suitable for subsequent electrochemical sensing applications.

### 3.3. CV Characterization of MG/Chi Composite Film-Modified GCE

To validate the feasibility of the proposed sensing strategy, a series of comparative cyclic voltammetry (CV) measurements were performed. The obtained results are presented in Figure 2B, which presents the CV curves of various electrode configurations in the presence and absence of target analyte (isazofos).

As shown in Figure 2B(a), no distinct redox peaks were detected upon scanning the bare glassy carbon electrode (GCE) in blank phosphate-buffered saline (PBS, pH 7.50). Similarly, the bare GCE exhibited no redox activity in 100 μM isazofos solution (Figure 2B(b)). This observation confirms that isazofos is intrinsically electrochemically inactive and cannot be directly detected at the bare GCE. In contrast, the MG/Chi composite film-modified GCE displayed well-defined redox peaks at approximately 0.42 V and 0.40 V in blank PBS (Figure 2B(c)), which attests to the intrinsic electrochemical activity of the methyl green (MG) moiety within the composite film. When the modified electrode was exposed to 100 μM isazofos (Figure 2B(d)), a pronounced decrease in peak current was noted relative to the blank PBS control. This decrease in electrochemical signal implies a specific interaction between MG and isazofos, which results in the attenuation of the redox response of the MG moiety in the composite film. These findings validate the feasibility of employing the MG/Chi composite film-modified GCE for the indirect detection of OPPs.

### 3.4. Influence of Scan Rate on Electrochemical Redox Response

**Figure 3:**
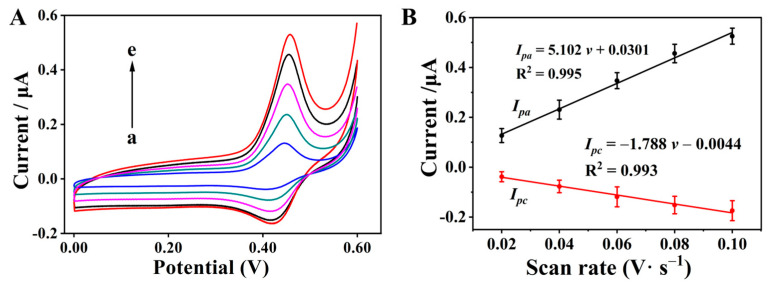
(**A**) Cyclic voltammetry (CV) curves of the MG/Chi composite film-modified electrode at different scan rates: (a) 0.02; (b) 0.04; (c) 0.06; (d) 0.08; (e) 0.10 V/s. (**B**) Linear plots of peak current versus scan rate.

To investigate the electrochemical kinetic characteristics of the MG/Chi composite film-modified electrode and identify the dominant control mechanism of its surface redox reaction, a series of CV measurements were systematically carried out over a controlled range of scan rates from 0.02 to 0.10 V s^−1^. The corresponding CV curves acquired at different scan rates are shown in Figure 3A, which clearly illustrates a gradual and linear enhancement in both anodic peak current (*i_pa_*) and cathodic peak current (*i_pc_*) with the incremental increase in scan rate (*v*).

For quantitative characterization of this linear relationship, linear regression fitting was performed by plotting the peak currents (*i_pa_* and *i_pc_*) as a function of the scan rate (*v*), and the resulting linear fitting equations for the anodic and cathodic redox peaks are given as follows:

Anodic peak: *i_pa_* = 5.102 *v* + 0.0301 (R^2^ = 0.995); Cathodic peak: *i_pc_* = −1.788 *v* − 0.0044 (R^2^ = 0.993)

where *i_pa_* and *i_pc_* are expressed in microamperes (μA), and *v* is in volts per second (V s^−1^); R^2^ represents the correlation coefficient of the linear fitting (Figure 3B). The high correlation coefficients (R^2^ > 0.99) for both anodic and cathodic peaks are indicative of a highly linear dependence of peak current on the scan rate, a key electrochemical signature that confirms the redox reaction at the MG/Chi composite film-modified GCE surface is predominantly adsorption-controlled. This kinetic behavior is in good agreement with the reported electrochemical characteristics of similar composite film-modified electrodes in the previous literature [45].

### 3.5. Proposed Sensing Mechanism for Isazofos Detection

The structural stability of the methyl green/chitosan (MG/Chi) composite film is dominated by three core intermolecular interactions. First, hydrogen bonding, the primary driving force for film stability, is formed between the -OH and -NH_2_ groups of chitosan (Chi) and the C=O and -NH- groups of methyl green (MG), as well as intra- and intermolecular hydrogen bonds within Chi molecular chains. Second, stable electrostatic attraction is generated between the positively charged protonated -NH_3_^+^ groups on the Chi backbone and the negatively charged -SO_3_^−^ groups on MG molecules, strengthening the intercomponent binding of the composite. Third, hydrophobic association between the aromatic ring structure of MG and the hydrophobic segments of Chi further enhances the interchain cohesion and structural compactness of the film. Fourier transform infrared (FTIR) spectroscopy characterization validated the above mechanism: the characteristic -OH stretching vibration peak of Chi at 3420 cm^−1^ showed a distinct red-shift in the MG/Chi composite spectrum, which arises from the formation of hydrogen bonds between MG and Chi, providing direct experimental evidence for the proposed intermolecular interactions.

**Figure 4:**
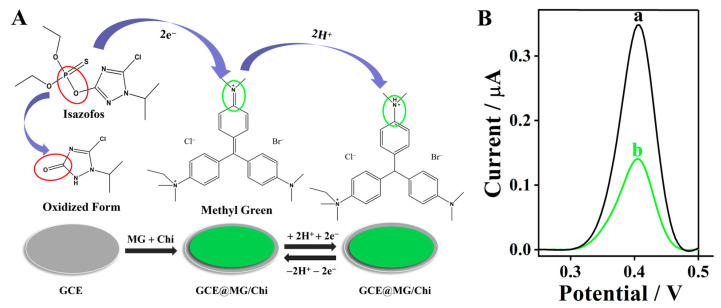
(**A**) Schematic illustration of the proposed reaction mechanism for isazofos detection using the MG/Chi composite film-modified electrode. (**B**) Differential pulse voltammetry (DPV) curves of GCE in PBS (pH 7.50). (a) The MG/Chi composite film-modified GCE; (b) MG/Chi composite film-modified GCE + 100 μM isazofos.

Based on the above experimental results and electrochemical characterizations, a plausible sensing mechanism underlying the detection of isazofos using the MG/Chi composite film-modified GCE is proposed herein. MG, serving as the electrochemically active moiety of the composite, is immobilized on the GCE surface via the Chi matrix, which acts as a stable biopolymer support. When the MG/Chi composite film-modified GCE is exposed to isazofos, a specific interaction takes place between the electrochemically active MG moiety and the target isazofos molecules. It is hypothesized that the quinone moiety of MG mediates the oxidation of the phosphorus–oxygen (P–O) bond within the isazofos molecule, as schematically illustrated in Figure 4A. “The P–O bond of isazofos undergoes oxidative cleavage under the experimental conditions, and isazofos acts as an electron donor in this redox reaction, releasing electrons that are directly transferred to the oxidized methyl green with a quinoid structure. The quinoid moiety of methyl green accepts the electrons and is reduced to a benzene ring structure with a non-conjugated system, which is electrochemically inactive and thus leads to the decrease in the voltametric response signal.” The residual unreacted MG moieties on the electrode surface still exhibit intrinsic redox activity at the same characteristic potential; however, a noticeable decline in the redox peak current is observed due to the reduced surface concentration of electrochemically active MG species. By quantifying the relative change in the MG redox peak current before and after the modified GCE is incubated with isazofos, a linear calibration curve for isazofos can be established, thereby enabling the quantitative determination of isazofos.

### 3.6. Differential Pulse Voltammetry (DPV) Response for Isazofos Detection

While CV is a powerful electrochemical tool for elucidating electrode reaction mechanisms, it suffers from limited sensitivity for the quantitative determination of low-concentration analytes. This limitation arises primarily from its relatively broad redox peaks, elevated background current, and overlapping signal components, which hinder accurate quantification of trace-level targets. To overcome these inherent drawbacks and facilitate sensitive, reliable detection of isazofos at trace concentrations, DPV was therefore adopted as the primary quantitative detection technique [45]. Compared with CV, DPV exhibits several distinct advantages, including superior detection limits, enhanced sensitivity, and improved peak resolution. These inherent merits enable DPV to generate well-resolved, sharp single-peak responses with minimal background interference, making it ideally suited for the precise quantitative analysis of isazofos in complex matrices. Based on preliminary CV characterizations, the anodic peak of methyl green (MG) was found to be better resolved and more electrochemically stable than its corresponding cathodic peak. Consequently, all DPV measurements were systematically performed over a potential window of 0.25 to 0.50 V (vs. Ag/AgCl reference electrode), specifically targeting the anodic redox response of MG. The representative DPV responses acquired under the various experimental conditions are depicted in Figure 4B, which demonstrate the feasibility of the developed MG/Chi-modified GCE for the detection of OPPs.

### 3.7. Optimization of Experimental Conditions

#### 3.7.1. Optimization of Methyl Green Concentration

**Figure 5:**
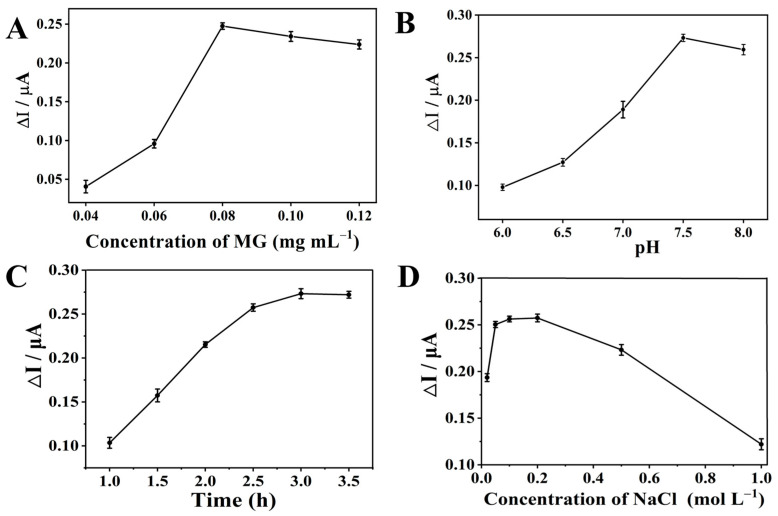
(**A**) DPV peak current change with various MG concentrations; (**B**) DPV peak current change at different pH values; (**C**) DPV peak current change with different coating times; (**D**) DPV peak current change with different ionic strengths.

As an electrochemically active material modified on the electrode surface, MG plays a crucial role in the electrochemical response toward the target analyte, and thus its concentration requires preliminary optimization. After conducting systematic screening experiments on various methyl green/chitosan (MG/Chi) composite film-modified materials, the corresponding results were acquired, as presented in Figure 5A.

Specifically, when the concentration of isazofos was fixed 100 μM, the peak current difference was found to increase with the gradual elevation of MG concentration in the composite film. Notably, the electrode modified with the 0.08 mg/mL MG/Chi composite film exhibited the maximum peak current difference, indicating the optimal MG concentration for electrochemical detection of OPPs. Thus, this concentration was selected for all subsequent experiments to ensure high sensitivity and reproducibility in the detection process.

#### 3.7.2. Optimization of Buffer pH

At pH < 5.0, Chi becomes highly protonated, resulting in intensified electrostatic repulsion; at pH > 8.5, the protonation of the -NH_2_ groups in Chi is weakened, accompanied by partial deprotonation of MG. Both scenarios induce the disintegration of the composite layer. Moreover, isazofos undergoes degradation under both acidic and alkaline conditions, making solution pH a critical parameter for the present experiment. A series of systematic experiments were thus carried out, and the corresponding experimental curve is presented in Figure 5B. As depicted in Figure 5B, a pH value of 7.5 was identified as the optimal experimental condition. This phenomenon can be rationalized by the solubility behavior of chitosan and the degradation characteristic of isazofos: chitosan shows favorable solubility in acidic media, which may induce partial detachment of the chitosan moiety from the modified material under acidic conditions; this structural instability is responsible for the relatively low peak current detected in acidic environments. By contrast, a pH of 7.5 not only ensures the structural integrity of the modified material (i.e., no component detachment) but also results in an extremely slow degradation rate of isazofos at this pH.

#### 3.7.3. Optimization of Coating Time

Coating time also serves as a critical factor governing the overall performance of the experiment. Insufficient coating time would result in incomplete drying of the modified material, which hinders its stable immobilization on the electrode surface. This issue not only contaminates the detection solution but also causes a significant deterioration in the detection response. By contrast, an excessively prolonged coating time may induce the detachment of the modified material or trigger unforeseen reactions with airborne substances. The optimization results for coating time are presented in Figure 5C. As illustrated in Figure 5C, the peak currents of the modified material coated for 3 h reached a saturated level. On this basis, 3 h was selected as the optimal coating time, and all subsequent experiments were conducted under this condition.

#### 3.7.4. Optimization of Ionic Strength

Ionic strength is a critical parameter governing the structural stability of the MG/Chi composite sensing film (whose integrity relies on electrostatic interactions and hydrogen bonding between MG and Chi) and the electrochemical performance of the fabricated sensor. We thus systematically evaluated the effect of ionic strength on the sensor response by adjusting the NaCl concentration in 0.1 M PBS (pH 7.5) from 0.01 to 1.0 M. As illustrated in Figure 5D, the sensor maintained a stable and optimal electrochemical response within the ionic strength range of 0.05–0.2 M, with peak current fluctuation controlled below 5%. However, excessively high ionic strength (NaCl concentration > 0.5 M) induced a strong electrostatic shielding effect: abundant free ions neutralized the surface charges of MG and Chi, disrupted their stable crosslinking network, and further caused swelling and partial exfoliation of the composite film on the GCE surface. This structural damage reduced interfacial electron transfer efficiency and triggered leakage of the electroactive component MG, ultimately leading to signal attenuation and deteriorated signal stability and reproducibility.

#### 3.7.5. Optimization of Drop-Coating Volume for MG/Chi Composite Solution

The coating thickness of the sensing layer exerts a critical impact on the analytical performance of the proposed electrochemical sensor. We therefore systematically investigated the effect of coating thickness (regulated via a drop-coating volume ranging from 1.0 to 20 μL) on the sensing performance of the sensor. Stylus profilometry measurements confirmed that the thickness of the MG/Chi composite film on the glassy carbon electrode (GCE) surface was 48.2 ± 3.5 nm under the optimal preparation conditions (MG concentration: 0.08 mg mL^−1^, Chi concentration: 5.0 mg mL^−1^, drop-coating volume: 5.0 μL), which was verified to deliver the optimal sensing performance. When the film thickness was below 48 nm, the electrochemical signal response increased monotonically with an increasing thickness, which is attributable to the elevated loading of electroactive MG sites immobilized on the electrode surface. Conversely, when the thickness exceeded 48 nm, the electron transfer resistance at the electrode–electrolyte interface increased significantly, resulting in an attenuation of the redox signal of MG and a subsequent decline in detection sensitivity.

Composite film microstructure and surface roughness characterization: AFM characterization corroborated that the MG/Chi composite sensing layer formed a uniform, continuous film without observable cracks, pinholes, or other microstructural defects. The root mean square roughness (Rq) of the coating surface was 1.86 ± 0.12 nm, and the arithmetic mean roughness (Ra) was 1.42 ± 0.09 nm, verifying the ultrasmooth surface of the as-prepared sensing layer. This smooth, defect-free microstructure not only confers robust mechanical and electrochemical stability to the composite film but also effectively mitigates non-specific adsorption of interfering components from the complex sample matrix at the sensing interface, which is identified as a key contributor to the outstanding anti-interference capability of the developed sensor.

### 3.8. Analysis Performance for Detection of Isazofos

#### 3.8.1. Performance Evaluation of the Sensing System

**Figure 6:**
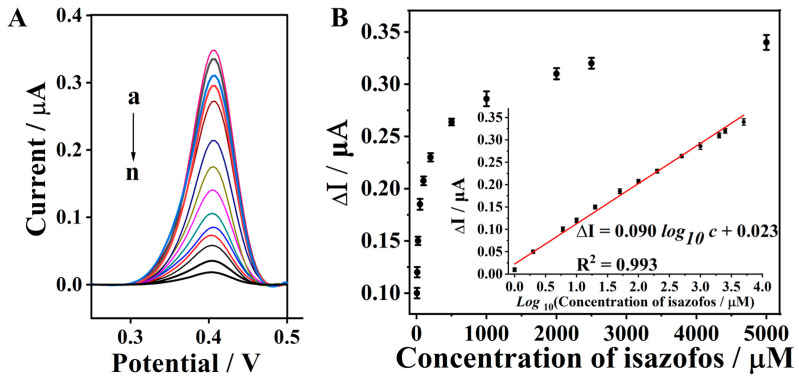
(**A**) Differential pulse voltammetry diagrams of the biosensing system in the presence of isazofos with different concentrations: (a) 0; (b) 1; (c) 2; (d) 6; (e) 10; (f) 20; (g) 50; (h) 100; (i) 200; (j) 500; (k)1000; (l) 2000; (m) 2500; (n) 5000 µM. (**B**) Calibration curve corresponding to the DPV peak current (at the potential of 0.42 V) as a function of isazofos concentration. Inset shows the linear relationship between the DPV peak current changes and the logarithm of the isazofos concentration ranging from 1 to 5000 µM. The preconcentration time was 6 min for all tests. The error bars represent the standard deviation of three tests.

To assess the analytical performance of the as-proposed sensing strategy, DPV responses toward isazofos at various concentration levels were recorded under the optimized experimental conditions. As depicted in Figure 6A, the DPV peak currents decreased progressively with an increasing isazofos concentration. With an increasing isazofos concentration, the methyl green immobilized on the electrode surface undergoes nearly complete reaction. Furthermore, peak current changes exhibited a robust linear relationship with the logarithm of isazofos concentration over the range of 2–2500 µM (the inset of Figure 6B). The regression equation is Δ*I* = 0.090 log_10_ *c* + 0.023, (*I*: DPV response currents, μA; *c*: isazofos concentration, μM), with the correlation coefficient of 0.993. The limit of detection (LOD) for isazofos was determined to be 0.60 μM at a signal-to-noise ratio of 3 (S/N = 3). This LOD is favorably comparable to or even lower than those of most previously reported isazofos sensing platforms, with a detailed performance comparison summarized in Table S1.

#### 3.8.2. Anti-Interference Assay

**Figure 7:**
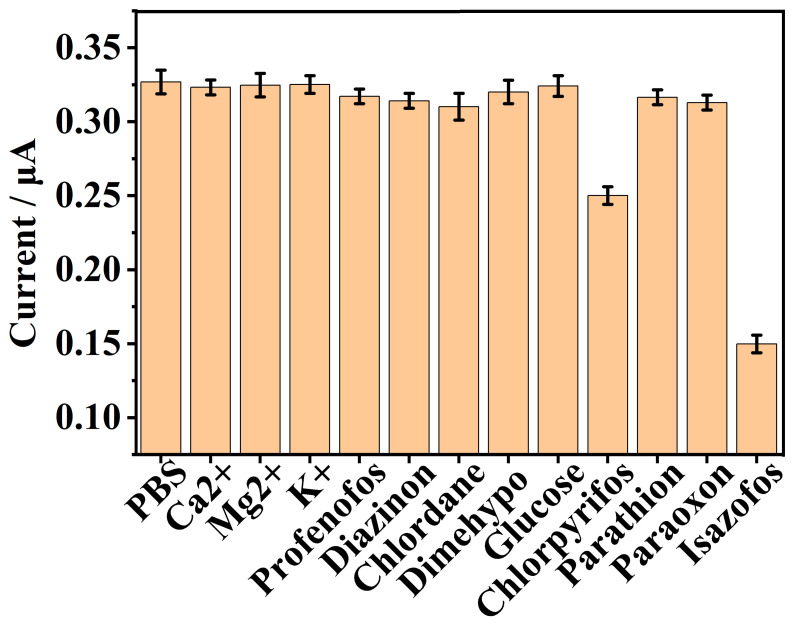
DPV currents of the biosensing system in the presence of Ca^2+^, Mg^2+^, K^+^, profenofos, diazinon, chlordane, dimehypo, glucose, chlorpyrifos, parathion, paraoxon and isazofos, respectively, in which “PBS” indicates the condition in the absence of pesticides or inorganic ions. The concentrations of the above pesticides and inorganic ions were all 1.0 mM, except the isazofos concentration, which was 100 µM. The error bars represent the standard deviation of three measurements.

To verify the specificity of the proposed homogeneous electrochemical strategy for isazofos detection, we further investigated its performance by measuring the electrochemical responses of the system toward potential coexisting substances and structural analogs of isazofos, including Ca^2+^, K^+^, Mg^2+^, profenofos, diazinon, chlordane, dimehypo, glucose, chlorpyrifos, parathion and paraoxon. As shown in Figure 7, a decreasing electrochemical signal was obtained only in the presence of the isazofos. In contrast, the DPV peak currents showed negligible changes in the presence of other interfering substances and were comparable to the background signal. We postulate that such behavior arises from the intrinsic molecular structure of isazofos, a prototypical thiophosphorus organophosphorus pesticide whose phosphorus center is covalently bound to a 5-chloro-1-isopropyl-1,2,4-triazol-3-yl substituent. The chlorine atom and triazole heterocycle collectively exert strong electron-withdrawing effects, which markedly diminish the electron-cloud density of the P–O bond and elevate its electrophilic reactivity. Relative to chlorpyrifos, parathion, and paraoxon, isazofos displays a more favorable steric configuration that exhibits excellent complementary fitting with the binding cavity of methyl green. This synergistic modulation of electronic characteristics and steric constraints reduces the activation energy of the corresponding reaction, confers the sensing system with a superior specific recognition capability toward isazofos, and thereby permits highly selective detection over other structural analogues. These results confirm that the proposed strategy exhibits excellent selectivity for isazofos against other interfering pesticides and inorganic ions. For the complete elimination of chlorpyrifos interference, we propose that this goal can be accomplished via the following design strategies in future research: (1) Modifying the sensing interface with isazofos-specific molecularly imprinted polymers (MIPs) to fabricate a selective recognition layer, thereby reducing the non-specific adsorption and reaction of structural analogs including chlorpyrifos. (2) Optimizing the composition and morphology of the electrode modification layer (e.g., introducing metal nanocomposites with catalytic selectivity) to regulate the electron transfer efficiency of the target analyte (isazofos) and enhance the anti-interference capability of the sensor. (3) Coupling the voltammetric sensor with pre-separation techniques (e.g., solid-phase extraction) for the pretreatment of real samples, so as to eliminate the interference from coexisting organophosphorus pesticides. Owing to such superior anti-interference performance, the as-proposed electrochemical assay for isazofos holds great potential for practical application in complex matrix samples.

#### 3.8.3. Reproducibility and Reusability of the Fabricated Electrochemical Sensor

To evaluate the electrode-to-electrode reproducibility of the proposed sensor, four parallel MG/Chi composite film modified glassy carbon electrodes (GCEs) were fabricated under strictly identical experimental conditions, and their differential pulse voltammetry (DPV) responses toward 100 µM isazofos were recorded. The relative standard deviation (RSD) of the obtained DPV signals was calculated to be 4.08%, demonstrating excellent reproducibility of the established electrode fabrication protocol. The long-term stability of the voltammetric sensor was evaluated by testing the voltammetric response to isazofos once a week under the optimal experimental conditions, with the sensor stored in a refrigerator at 4 °C for standby between tests. The results demonstrated that the average response of a single electrode still retained 89.0% of its initial value after 21 days (Figure S1).

For batch-to-batch reproducibility assessment, three independent batches of the modified electrodes were prepared on separate days, and their DPV responses to 100 µM isazofos were measured under consistent testing parameters. An RSD of 4.56% was obtained for the detection signals, verifying the outstanding robustness of the sensor fabrication method and its high suitability for scaled-up batch preparation.

The reusability of the as-fabricated modified electrode was further investigated via consecutive DPV detection of 100 µM isazofos. After each measurement, the electrode regenerated via cyclic voltammetry scanning in blank PBS buffer (scan rate: 50 mV/s, potential window: 0.0 V to 0.6 V) for 10 cycles, which can completely remove the adsorbed target analyte and reaction by-products on the electrode surface, and restore the initial electroactivity of the modified electrode. The results showed that the sensor maintained a stable electrochemical performance without significant signal attenuation after nine consecutive detection cycles, with the DPV response remaining above 87% of its initial value. When the number of detection cycles exceeded nine, the DPV signal dropped rapidly to less than 70% of the initial response (Figure S2). This signal decay was mainly attributed to the progressive desorption of MG from the Chi matrix, as well as mild fouling of the electrode surface after repeated exposure to the detection system. Notably, for the electrode after 10 detection cycles, a simple recoating with a small volume of MG solution (2.0 μL, 1 mg/mL) could restore the sensor response to more than 80% of its initial signal, which greatly improves the practical application potential of the proposed sensor.

### 3.9. Analysis of Isazofos in Actual Samples

Isazofos is a dedicated organophosphate insecticide targeting soil-dwelling pests, with its residues predominantly accumulating in root and tuber agricultural products. Therefore, ginger and carrots were purchased from local markets, and their pesticide residues were analyzed and quantified following sample pretreatment. OPPs in natural water samples collected from Hongzi Lake in the Qingdao Agricultural University were also investigated. Experimental results demonstrated that the levels of OPPs in these samples were extremely low, falling below the limit of detection (LOD) of the developed sensing method. Thus, the standard addition method was employed for method validation. We spiked 20 μM and 100 μM isazofos standard solutions into the pretreated solution, and the resulting mixtures were diluted to 10 mL prior to analysis. The aforementioned experimental procedures were then repeated, with the corresponding results presented in Table 1.

## 4. Conclusions

A facile label-free electrochemical sensor based on MG/Chi composite-modified GCE was developed for selective and sensitive detection of the organophosphorus pesticide isazofos. Electrochemical characterizations (CV, EIS, DPV) confirmed the successful sensor fabrication, its adsorption-controlled redox behavior, and the specific MG-isazofos interaction. Under optimized conditions, the sensor showed a linear response to isazofos of 2.0–2500 µM, a limit of detection of 0.60 μM (S/N = 3), excellent anti-interference performance and good specificity. Spiked recovery tests in real samples (ginger, carrot, lake water) verified its practical reliability, with 93.6–108.5% recoveries and RSDs < 5.56% (n = 6). Compared with chromatographic techniques and commercial rapid kits, this sensing strategy exhibits clear advantages in cost and analysis time (detailed cost evaluation is provided in SI), highlighting its promise and potential for low-cost, on-site isazofos residue monitoring. This low-cost, easy-to-fabricate sensor with a stable performance provides a novel, effective approach for isazofos detection, and it holds great potential for on-site OPP monitoring in food safety and environmental analysis.

## Figures and Tables

**Table 1:** Precision (RSD %) and accuracy (recovery %) of isazofos spiked into the actual samples.

Sample	Isazofos Added (μM)	Measured (nM)	RSD (%) (n = 6)	Recovery (%)
Ginger	20	19.24	4.80	96.20
	100	108.50	4.37	108.50
Carrots	20	19.88	3.92	99.40
	100	102.43	4.08	102.43
Lake water	20	18.72	5.56	93.60
	100	98.15	5.00	98.15

## Data Availability

Data will be made available on request.

## Supplementary Materials

The following supporting information can be downloaded at: https://www.mdpi.com/article/10.3390/bios16040208/s1, Table S1: Comparison of various organophosphorus pesticide residue assay methods; Figure S1: The voltametric response of the system with 100 µM isazofos based on 21 days (3 weeks) detections; Figure S2: The voltametric response of the system with 100 µM isazofos based on 11 times repeat detections. References [46,47,48] are cited in the supplementary materials.
