# Supplementary material for: Novel Electrochemical Sensing Strategy for Organophosphorus Pesticide Residues
Source: Biosensors (Basel). 2026 Apr 7;16(4):208. doi: 10.3390/bios16040208 (PMC13115342; doi:10.3390/bios16040208)
Supplement: Supplementary file 1 [file biosensors-16-00208-s001.zip › biosensors-4174491-SI.pdf]

Supporting Information

Novel Electrochemical Sensing Strategy for Organophosphorus Pesticide Residues

Mingzhuo Xu 1,†, Chen He 2,†, Jiajing Zhang 1, Hao Yang 1 and Xiuzhong Wang 1,*

[1] College of Chemistry and Pharmaceutical Sciences, Qingdao Agricultural University, Qingdao, 266109，China

[2] Shandong Provincial Metrology and Testing Center, Jinan, 250102, China.

* Correspondence: xzwang@qau.edu.cn;

† These authors contributed equally to this work.

Table S1. Comparison of various organophosphorus pesticide residue assay methods

Target	Biosensing method	linear range	LOD	References
Chlorpyrifos	Colorimetric	3.33–1.33×10^3 μg L^{-1}	3.33 μg L^{-1}	[1]
Parathion Methyl	Colorimetric/fluorescent	8–1000 μg·L^{-1}	0.476 μg L^{-1}	[2]
Malathion	Electrochemical	1–200 μM	1.79 μM	[3]
Triazophos	Fluorescent	0.1–1.5×10^3 μg L^{-1}	0.058 μg L^{-1}	[4]
Chlorpyrifos	Electrochemical	1–150 μM	5.79 nM	[5]
Methyl Parathion	Electrochemical	0.1–100 mg L^{-1}	0.716 μg L^{-1}	[6]
Isazofos	Electrochemical	2–2.5×10^3 μM	0.60 μM	This work

References:

1. Zou, R.; Huang, Z.; Li, M.; Lin, Q.; Zheng, H.; Wang, J.; Yan, X.; Jin, R.; Li, H., Lab in an aerogel kit: Fe-g@sa-based aerogel sensor with high-performance peroxidase activity for chlorpyrifos pesticide detection. Biosensors and Bioelectronics 2026, 296, 118360.
2. Yan, K.; Wang, C.; Zhang, Y.; Li, Y.; Jin, Y.; Jiang, J.; Liu, S.; Zhan, J.; Yu, W.; Dong, X., et al., Protein-stabilized gold nanoclusters for colorimetric/fluorescence dual-mode detection of organophosphorus pesticides. FOOD CHEMISTRY 2026, 498.
3. Singh, K.R.; Singh, P.; Pandey, S.S., Highly efficient and selective biosensing of malathion utilizing bioengineered nanoplatform based on fe3o4. Langmuir 2026, doi: 10.1021.
4. Li, S.; Wang, Y.; Liu, H.; Sun, B., Covalent organic polymer cascade mno2nanosheets-based fluorescence-colorimetric dual-mode sensing system for highly sensitive detection of organophosphorus pesticides. Spectrochimica Acta. Part A, Molecular and Biomolecular Spectroscopy 2026, 350, 127431.
5. Huai, W.; Chu, H.; Qin, S.; Gao, L.; Han, S.; Zong, W.; Zhao, M., Construction of an electrochemical sensor based on mxene@ni3s2 "sandwich layer" nanomaterial and its ultra-sensitive detection of chlorpyrifos. MICROCHEMICAL JOURNAL 2026, 220.
6. Zhang, Z.; Ma, H.; Mo, H.; Zhu, N., Organophosphorus pesticide photoelectrochemical/electrochemical dual-mode smartsensors derived from synergistic co,n-tio2@zro2/3dgh platform. CHEMOSENSORS 2025, 13.

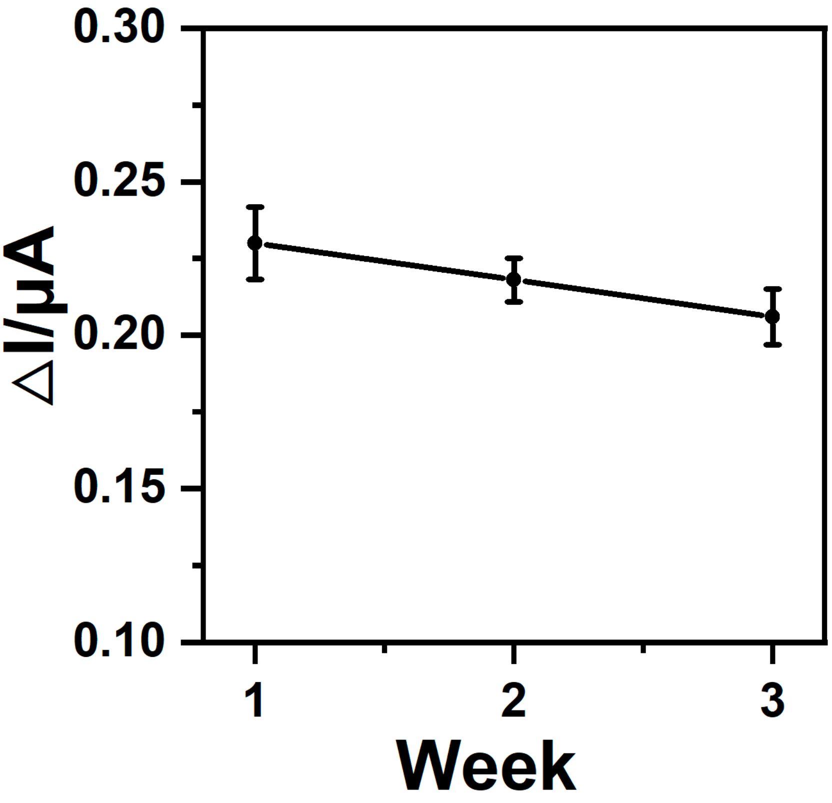

Figure S1 The voltametric response of the system with 100 μM isazofos based on 21 days (3 weeks) detections

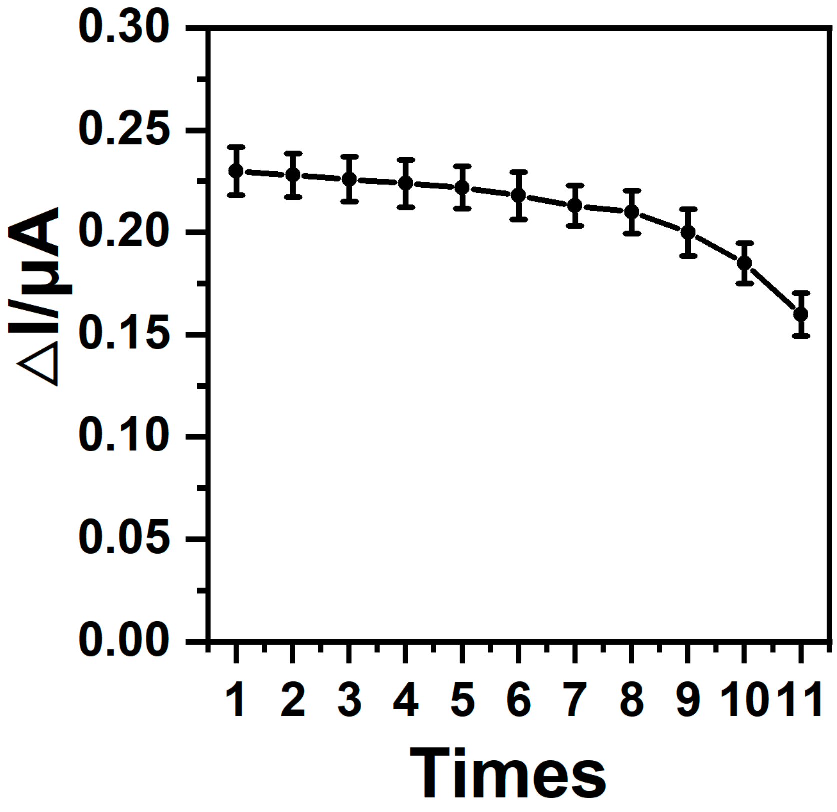

Figure S2 The voltametric response of the system with 100 μM isazofos based on 11 times repeat detections

Basic economic discussion of the sensing strategy

1. **Material cost per sensor**: The core materials for the MG/Chi composite film-modified glassy carbon electrode include methyl green, chitosan, and routine consumables (e.g., buffers, electrolytes). Based on actual experimental accounting, the total material cost for a single modified electrode is approximately 0.8–1.2 CNY (0.11–0.17 USD). All reagents are low-cost and commercially available.
2. **Reusability**: The modified electrode exhibits satisfactory stability. After storage at 4 °C, a single electrode can be reused for at least 9 valid detections of isazofos, leading to an amortized per-test material cost as low as 0.08–0.12 CNY (0.01–0.015 USD).
3. **Analysis time**: Without complex sample pretreatment or labeling steps, only simple extraction and dilution are required for real samples (ginger, carrot, lake water). The total time from sample preparation to final result is less than 15 minutes, enabling rapid on-site screening.
4. **Comparison with mainstream methods**:

   Chromatographic methods (HPLC, GC-MS): ~50–100 CNY per test, 1–2 h per analysis, requiring expensive instruments and skilled personnel.

   Commercial rapid test kits: ~3–5 CNY per test, 20–30 min per analysis.

   In contrast, our method exhibits obvious advantages in both cost and time efficiency, while maintaining high sensitivity (LOD = 0.60 μM) and satisfactory accuracy (recoveries: 93.6–108.5%), which provides solid quantitative support for our "low-cost" claim.
